# Dr. Newton Shaffer (1846-1928): Renowned Orthopedic Surgeon and Founder of the American Orthopaedic Association

**DOI:** 10.7759/cureus.60529

**Published:** 2024-05-17

**Authors:** Cameron Gerhold, Renish N Contractor, Michael J Sweeney

**Affiliations:** 1 Department of Orthopedic Surgery, Florida State University College of Medicine, Tallahassee, USA; 2 Department of Urology, Florida State University College of Medicine, Daytona Beach, USA; 3 Department of Clinical Sciences, Florida State University College of Medicine, Tallahassee, USA

**Keywords:** medical history, newton shaffer, american orthopaedic association, orthopaedic surgery, historical vignette

## Abstract

An unsung hero of American orthopedic surgery is the largely forgotten Dr. Newton Melman Shaffer (1846-1928). Upon graduating from medical school at New York University, Shaffer began his career training at the Hospital for the Ruptured and Crippled in 1867. Shaffer then went on to practice at St. Luke’s Hospital and New York Orthopaedic Dispensary and Hospital where he became chief. Here, Shaffer made major contributions to the field in treating clubfoot and tuberculosis. He then declared orthopedics as a separate entity from general surgery at the 10th International Medical Congress. He helped start the American Orthopaedic Association to push for the recognition of American orthopedics to the international community. In 1900, Shaffer opened the first state-run hospital for underprivileged children requiring rehabilitation. During his career, Shaffer advocated for conservative orthopedic treatments, aided in the invention of medical devices, contributed largely to academic orthopedics, and successfully advocated for the inception of the field of orthopedic surgery.

## Introduction and background

Early life and education

Newton Melman Shaffer, the son of James Newton Shaffer, a minister, and Jane Emeline Hale, was born on February 14, 1846, in Kinderhook, New York [1]. Newton Shaffer grew up with four siblings: Wilbur Shaffer, Anna Shaffer, Ella Shaffer, and Edward Livingston Shaffer. He was an avid fisherman, among other hobbies. Newton Shaffer married Margaret Hyde Perkins from Gardiner, Maine, with whom he had one son, Newton M. Shaffer Jr.

At the age of 21 years, he received his medical degree from New York University in 1867. Soon after, he began his career as an orthopedic surgeon at the Hospital for the Ruptured and Crippled, the oldest orthopedic hospital in the nation, in New York City. At the Hospital for the Ruptured and Crippled, Shaffer was trained by James Knight and remained on staff there until 1870 [2]. It is widely believed that Shaffer chose to pursue a career in orthopedic surgery because of his apprenticeship with Knight since Knight spent much of his time developing custom braces and gadgetry for his patients who were in desperate need of orthopedic care [2].

## Review

Career as an orthopedic surgeon and surgeon-in-chief

**Figure 1:**
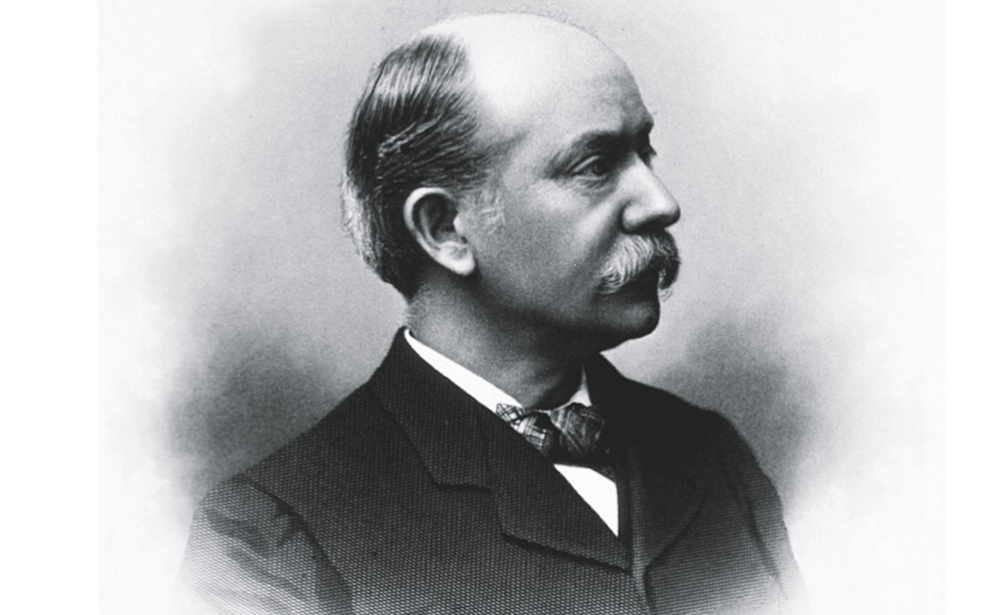
Still portrait of Newton M. Shaffer. Credit: Permission obtained from The Ridgefield Press [1].

Shaffer began his career as an orthopedic surgeon in 1870 at St. Luke’s Hospital, the first orthopedic service in a general hospital (Figure 1) [3]. Shaffer’s work in orthopedic surgery caught the attention of Theodore Roosevelt Sr., a philanthropist and father of President Theodore Roosevelt Jr., who had helped start the New York Orthopaedic Dispensary and Hospital. In 1871, under the guidance of Roosevelt Sr., Shaffer concurrently began working at the New York Orthopedic Dispensary and Hospital (currently known as New York Orthopaedic Hospital, which is affiliated with the Columbia University-Presbyterian Medical Center), where he succeeded Charles Fayette Taylor as chief [1,4]. In addition to his role as a hospital chief, he established the orthopedic service at St. Luke’s Hospital in New York City in 1872 with the support of Theodore Roosevelt Sr. [2,4]. This was a notable accomplishment since it is likely the first instance in the United States of a major hospital establishing an orthopedic department independent of control by the hospital’s chief of surgery, setting the stage for more hospitals to follow this direction [2].

Shaffer’s specialty was children, particularly those whose bones had been ravaged by tuberculosis [2]. Working for St. Luke’s provided Shaffer with the opportunity to participate in a wide variety of procedures, including one surgical correction, which was ultimately named after him: Shaffer’s nondeforming clubfoot [2,4]. Shaffer would go on to publish a paper describing the anatomy of nondeforming clubfoot and successful surgical treatment techniques for this ailment, a paper that some consider to be one of his most important contributions to medicine [2]. Nondeforming clubfoot was a term coined by Shaffer, who realized that in either patients who were paralyzed in both lower extremities or in patients who had unilateral paralysis, abnormalities were present in the less affected lower limb [5].

While serving as chief of staff at New York Orthopaedic Hospital and directing orthopedic services at St. Luke’s, Shaffer also became a distinguished orthopedics professor at New York University and Cornell Medical School [1,2]. Shaffer served as the chief of the New York Orthopaedic Dispensary and Hospital until 1891.

In addition to being a prominent entity in the New York medical community, Shaffer had strong connections worldwide that allowed him to make impactful changes in the field of orthopedic surgery. In 1890, Dr. Shaffer attended the 10th International Medical Congress in Berlin [2,6], a meeting that would forever change the trajectory of orthopedics. Ultimately, Shaffer’s goal in attending was to proclaim orthopedic surgery as a separate entity from general surgery. He wrote a book entitled *What Is Orthopaedic Surgery?: Read Before the Orthopaedic Section of the Tenth International Medical Congress, Berlin, August 5, 1890*, in which he describes the pioneers and history of orthopedics while emphasizing the need for orthopedics to be declared separate from general surgery [6]. Within this book, he details personal accounts of patients with physical deformities being turned away by general surgeons and sent to physicians who focused solely on deformities instead, like himself. His overall message was that individuals affected by deformities and crippling injuries should be treated by physicians who were passionate about these issues, as this would provide patients with the most conservative and thoughtful treatment while improving their outcomes [6]. In this book, he states: “If he possesses mechanical tastes and ability and devotes himself to orthopedic work for a sufficient period, he will almost surely succeed in reaching a high place. But if he attempts simultaneously to do the work that would naturally fall to the general surgeon, he will, sooner or later, become the latter in effect, if not in name” [6]. Ultimately, the 10th International Medical Congress concluded by accepting orthopedic surgery as a recognized specialty worldwide [2].

In 1900, Shaffer continued to spread his love for orthopedics and ideas surrounding the conservative management of orthopedic ailments by opening the New York State Hospital for Crippled and Deformed Children [1,7]. This hospital was founded partially due to the political support provided by Governor Theodore Roosevelt, who was serving as the 36th governor of New York at the time [1]. Only two years later, Shaffer would gain notability for assisting Roosevelt, a prominent political figure both then and now, with his own orthopedic injury [1]. Roosevelt sustained a leg fracture when a trolley hit his horse-drawn carriage, and this resulted in an infection that necessitated several months of recovery [1]. After hearing of the unfortunate incident, Shaffer wrote the president to inform him that he built a special chair that would allow him to easily carry up flights of stairs [1].

These events established Shaffer as one of the most prominent figures in orthopedic surgery worldwide and one of the largest advocates of the profession.

Founding of the American Orthopedic Association

In 1887, Dr. Shaffer and Dr. Virgin P. Gibney founded the American Orthopedic Association (AOA) [8,9]. Dr. Shaffer strongly pushed for its development to call together orthopedic surgeons in the United States and to push for the recognition of American orthopedic surgery and its practices in Europe [8]. This is the first orthopedic association to provide orthopedic surgeons the opportunity to convene and discuss important issues within their field. The first AOA Annual Meeting took place on June 15, 1887, at the New York Academy of Medicine, New York, where over 30 chapter members were present and the Constitution and Bylaws of the AOA were established [8]. Virgil P. Gibney would become the first-ever president of the AOA, with Newton Shaffer becoming the second [8]. As the oldest and most distinguished orthopedic association, the AOA has had significant involvement in developing other major orthopedic associations. Since its inception, the AOA has aided in the growth and founding of the American Academy of Orthopaedic Surgeons (founded in 1933), the American Board of Orthopaedic Surgeons (1934), and the Orthopaedic Research and Education Foundation (founded in 1953) [8].

Legacy and impact

The New York State Hospital for the Care of Indigent Crippled and Deformed Children, which Shaffer established in 1900, was one of the first state-run freestanding rehabilitation hospitals. It offered services to children in New York State whose parents were not able to pay for private treatment and those ravaged by tuberculosis [10]. While caring for these patients, Shaffer stressed the patient and conscientious application of treatment techniques for orthopedic injuries to correct deformities by applying bracing, exercise, and traction to promote the recovery of these children [11]. The hospital is now known as the Helen Hayes Hospital and continues to provide rehabilitative care for individuals of all ages with a wide array of musculoskeletal and neurological conditions.

Shaffer authored numerous orthopedic articles and books, such as *Hysterical Elements in Orthopaedic Surgery* (1880), where Shaffer describes the muscular elements in joint diseases. Shaffer’s contributions in this piece brought attention to pathological conditions that, up to this point, had been unknown and, in part, ignored. Additionally, Shaffer authored other works such as *Prognosis and Treatment of Ankle-joint Disease* (1882), *Brief Essays on Orthopedic Surgery: Including a Consideration of Its Relation to General Surgery, Its Future Demands* (1898), *On the Cause and Mechanical Treatment of Subluxation of the Semilunar Cartilages of the Knee-Joint* (1898), *Pott's Disease, Its Pathology and Mechanical Treatment* (1923), and *Selected Essays on Orthopaedic Surgery* (1924). These works, in addition to his other contributions to the field of orthopedic surgery, Shaffer’s founding of the AOA, his time spent serving as the Executive Director of the Congress of American Physicians and Surgeons, his academic contributions to orthopedics, and his advocacy for the separation of orthopedics from general surgery have allowed for the successful development of orthopedic surgery as a profession.

After leaving a tremendous impact on medicine as we know it, Newton Shaffer passed away on January 2, 1928, in New York, NY, at the age of 82 years [12].

## Conclusions

Dr. Newton Melman Shaffer not only made a profound impact on the lives of his patients, many of whom suffered greatly due to their orthopedic ailments, but also left an indelible mark on the medical community. After establishing orthopedics as a unique, distinct field of medicine at the 10th International Medical Congress, he founded the AOA. The AOA is still a well-known organization today, functioning to address critical issues within the field, excel in orthopedic education, and develop orthopedic leadership. Without Newton Shaffer, orthopedics would likely still fall under the realm of responsibilities of general surgery, leaving no physicians trained solely devoted to the prevention, diagnosis, and treatment of disorders and diseases of the joints, tendons, ligaments, muscles, and bones.
